# Reconstruction of non-uniformly sampled five-dimensional NMR spectra by signal separation algorithm

**DOI:** 10.1007/s10858-017-0095-8

**Published:** 2017-02-28

**Authors:** Krzysztof Kosiński, Jan Stanek, Michał J. Górka, Szymon Żerko, Wiktor Koźmiński

**Affiliations:** 1 Faculty of Chemistry, Biological and Chemical Research Centre, University of Warsaw, Żwirki i Wigury 101, 02-089 Warsaw, Poland; 2 Faculty of Physics, Division of Biophysics, Institute of Experimental Physics, University of Warsaw, Pasteura 5, 02-093 Warsaw, Poland

**Keywords:** High-dimensional NMR, 5D, Non-uniform sampling, Spectral reconstruction, Signal separation algorithm, Intrinsically disordered proteins, Resonance assignment

## Abstract

A method for five-dimensional spectral reconstruction of non-uniformly sampled NMR data sets is proposed. It is derived from the previously published signal separation algorithm, with major alterations to avoid unfeasible processing of an entire five-dimensional spectrum. The proposed method allows credible reconstruction of spectra from as little as a few hundred data points and enables sensitive resonance detection in experiments with a high dynamic range of peak intensities. The efficiency of the method is demonstrated on two high-resolution spectra for rapid sequential assignment of intrinsically disordered proteins, namely 5D HN(CA)CONH and 5D (HACA)CON(CO)CONH.

## Introduction

In recent years remarkable progress has been achieved in the field of nuclear magnetic resonance spectroscopy (NMR) methodology. Investigation of complex biomolecules necessitates the use of at least three-dimensional spectroscopy to ensure sufficient spectral resolution. An essential technique to record four (and higher) dimensional spectra in reasonable experimental time is non-uniform sampling (NUS) (Nowakowski et al. 2015). A particularly successful approach is randomized sparse sampling which enables robust reconstruction of high-resolution 3D–4D spectra from a tiny fraction of data points compared to uniform sampling (Mobli and Hoch 2008; Coggins et al. 2010; Freeman and Kupče 2012; Hiller and Wider 2012; Kazimierczuk et al. 2012; Hassanieh et al. 2015).

Several strategies were proposed for high fidelity spectral reconstruction of sparsely sampled data sets. The most powerful and frequently used are maximum entropy reconstruction (Robin et al. 1991), multidimensional decomposition (MDD) (Orekhov and Jaravine 2011), compressed sensing (CS) (Kazimierczuk and Orekhov 2011; Holland et al. 2011), CLEAN (Högbom 1974; Coggins and Zhou 2008) and related signal separation algorithm (SSA) (Stanek and Koźmiński 2010; Stanek et al. 2012), SCRUB (Coggins et al. 2012) and SMILE (Ying et al. 2016). These methods are suitable for various kinds of NMR experiments, however, until now, they are all limited to either 3D or 4D spectroscopy due to high computational cost and/or memory limitations.

The recently developed NUS-based 5D experiments proved their utility (Haba et al. 2013; Piai et al. 2016; Baias et al. 2017) in particular for intrinsically disordered proteins (IDP). In this case fast conformational dynamics result in a high degree of chemical shift averaging, increasing spectral overlap and ambiguity of NMR assignment. Native 5D spectra resolve these degeneracies, however, until now they could only be obtained by non-uniform (i.e., zero-augmented) Fourier transformation (nuFT), realized by sparse multidimensional Fourier transform (SMFT) (Kazimierczuk et al. 2009).

An alternative to the acquisition of high dimensional spectra is projection spectroscopy (Nagayama et al. 1978). This approach evolved from simple applications (Szyperski et al. 1993) to an automated procedure (Hiller et al. 2007) and enabled to obtain information on up to 7 frequencies in a single experiment (Narayanan et al. 2010). However, severe peak overlap in projection spectra may hamper unambiguous peak assignment. A combination of both NUS and projection spectroscopy allows to preserve necessary resolution in projections and record seven frequencies in a single experiment (Żerko and Koźmiński 2015).

Incomplete sampling of the time domain is the reason for spectral artefacts. In the simplest case of nuFT of data sampled randomly without any restrictions, signal-to-artefact (S/A) ratio increases proportionally to the square root of the number of acquired points (Kazimierczuk et al. 2009). This unfavourably slow scaling of S/A with experiment time renders nuFT less effective and generally inferior to reconstruction methods. The amplitude of artefacts and their distribution are determined by sampling schedule and peak frequencies, and, most importantly, increases with spectral crowding.

Here we present a modified SSA algorithm and its efficient implementation, capable of reconstructing five-dimensional spectra. We demonstrate that artefact suppressed 5D spectra can be obtained within reasonable processing time and using limited memory and disk resources.

## Outline of the signal separation algorithm

The computational approach used in this work is an extension of the signal separation algorithm (SSA) previously described by Stanek et al. (Stanek and Koźmiński 2010; Stanek et al. 2012). Five dimensional data pose a particular challenge, since a reconstructed full spectrum or time-domain data does not typically fit in random-access or permanent memory of a standard computer. Additionally, Fourier transformation of an entire spectrum, even if performed in a block-by-block fashion, would require impractically long processing time. The first difficulty can be tackled by adapting the data structures used in SSA to represent localised spectral features. The solution to the second problem derives from the idea of SMFT, that is to employ a lower-dimensional experiment to identify relevant spectral regions prior to the analysis of a 5D spectrum. This allows us to analyse only local spectral features. A similar approach was presented for linear prediction algorithms (Tang and Norris 1988). In the following sections, we outline the modified algorithm and describe in more detail the employed data structures and memory-conserving techniques.

**Fig. 1:**
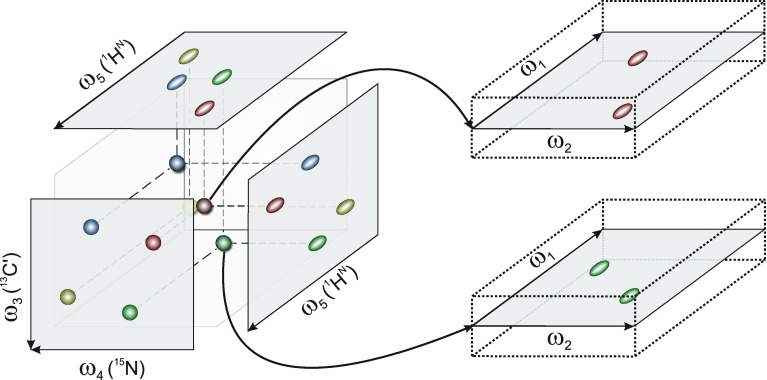
Peak search area in 5D SSA is determined by predefined two-dimensional cross-sections, which are computed based on peak positions in the 3D base spectrum. The vicinity of cross-sections is also explored, effectively the approach is tolerant to small variations of peak positions in dimensions ω_3_–ω_5_ with respect to the base spectrum (for simplicity, ω_3_–ω_5_ are compacted to one axis perpendicular to the ω_1_–ω_2_ cross-sections on the *right*)

SSA is an iterative algorithm based on the zero-augmented Fourier transform that progressively identifies spectral signals, temporarily removes them to enable detection of less intense features, and finally merges all information found. The core procedure consists of the following steps:

1. Prepare a 3D peak list sharing three dimensions (the directly sampled dimension ω_5_ and any two other, e.g. ω_3_, ω_4_) with the reconstructed spectrum. This list will be used to seed the algorithm with areas that contain strong peaks.
2. Fourier transform the 5D data in the directly sampled dimension (ω_5_). All subsequent steps are performed with ω_5_ already in the frequency domain. This in an optimization that does not materially affect the meaning of the other steps of the algorithm.
3. For each point in ω_5_, estimate the standard deviation of effective noise in directions perpendicular to ω_5_. This is done by computing the Fourier transform for a small, randomly chosen set of indirect dimension blocks at that point in ω_5_ and obtaining the median of spectral intensity in this set of blocks. Since the spectrum is very sparse, this procedure will ignore peaks and give a value which is a good approximation of the median in areas without any peaks, which in turn has a simple relation with the standard deviation for a normally-distributed random variable.
4. Divide the spectrum into small 5D blocks of equal size. Create a list of blocks which contain spectral points at the positions of peaks from step 1 in ω_3_–ω_5_. A small discrepancy in the positions of peaks is tolerated, since their vicinity in ω_3_–ω_5_ (up to a block size) will be analysed (see Fig. 1).
5. Find intense points in the spectrum by scanning the Fourier transformed blocks from step 4 one-by-one. Any point is considered intense if it has an absolute value higher than a predefined multiple of the standard deviation of effective noise (typically 10) and is not a part of a previously marked peak. Stop if no new points are found.

The threshold for peak detection can be rationalised as follows. In a model case of nuFT of a noiseless signal sampled randomly, S/A of 10 is obtained for as little as 50 samples. The vast majority of NMR experiments have to be sampled more extensively due to limited sensitivity, thus the requirement of minimum S/N = 10 does not prevent the detection of at least the strongest peaks. The only pitfall of this threshold is the case of extremely short acquisition of very sensitive NMR signals, where NUS artefacts dominate completely and no signal can initially be recognised.

On the other hand, the threshold should prevent the misinterpretation of noise spikes as signals, since this does not lead to any reduction in artefact level. Although SSA can eliminate spurious peaks in later iterations, performance is adversely affected. The default threshold ensures a negligible likelihood of false positive detection from a normally distributed noise $\left( erfc\left( \frac{10}{\sqrt{2}} \right)\approx 1.5\times {{10}^{-23}} \right)$ as well as if the noise distribution shows considerable deviations. Our experience shows that decreasing the threshold below 8 does not improve the quality of the final result, but considerably degrades performance, since in the final iterations, the residual signal is dominated by thermal noise.

6. Mark and subtract peaks starting from points found in step 5:

- Transform the current residual FID signal to obtain the spectral block with the intense point. Perform a bucket fill, marking all points adjacent to the peak and higher than a predefined multiple of the standard deviation of effective noise (this threshold is lower than for intense points, with a default value of 5).
- Find a time-domain signal whose Fourier transform approximates the marked spectral area of the peak by iterative modifications and inverse FT of a five-dimensional spectral peak model. The model is a set of points in the 5D frequency domain that corresponds to the marked area of the peak, but the intensities are adjusted to account for the non-linearity of the nuFT procedure. It is worth noting that this step does not make any use of the three peak coordinates provided in the input data; these are only used to inform where to search for peaks. Comprehensive discussion of this signal simulation procedure was presented in a previous work (Stanek and Koźmiński 2010).

7. Store the five-dimensional spectral model for later refinement and subtract its time domain representation from residual FID. Consequently, NUS artefacts stemming from this peak are also removed from the spectrum.
8. Once all points found in step 5 are processed, refine each peak by adding an inverse FT of its 5D spectral model (in the time-domain) back to the residual FID, and repeating the bucket fill and signal approximation procedure. Details of the refinement procedure are given in our previous publications (Stanek and Koźmiński 2010; Stanek et al. 2012). When finished, go to step 3.

The peak list used in step 1 is typically prepared using a 3D spectrum sharing the directly sampled dimension and any two other with the 5D spectrum. Since only artefacts stemming from the marked peaks can be removed by 5D SSA, one should carefully pick all visible peaks in a 3D spectrum even if they do not arise from the desired coherence pathways. The limitation of our approach is that a 5D experiment may show additional peaks due to limited phase cycling, however, they are usually weak and do not contribute significant artefacts to the effective noise.

In step 6, since the peaks are not allowed to overlap, the program needs to store the assignment of spectral points to peaks. This data is called the peak map. Note that the bucket fill procedure is implemented in such a way that peaks never shrink. In other words, if a given point belonged to the peak in iteration *i*, the same is guaranteed in iteration *i* + 1. Once the procedure is complete, the peak information and residual signal are stored on disk. Using this data, any arbitrarily chosen 2D cross-section (not restricted to the peak list used in step 1), as well as the 2D projections of the entire 5D spectrum, can be calculated using SMFT.

## Implementation of 5D SSA

**Fig. 2:**
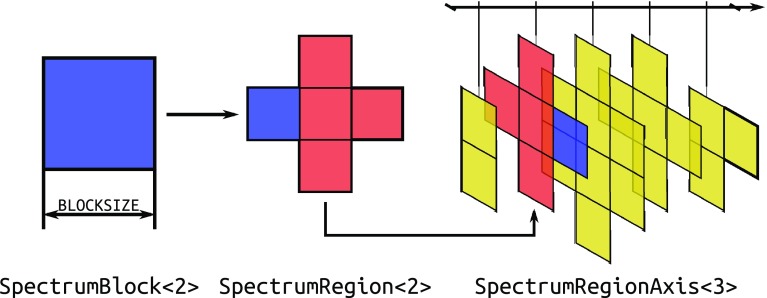
Sparse data structure used to represent isolated peak spectra. The fundamental component is *SpectrumBlock*, a contiguous (N−1)-dimensional block of the Fourier-transformed spectrum with a fixed size in each dimension. These blocks are used as values in *SpectrumRegion*, a binary search tree keyed by the minimum point of the block (i.e., the point with minimum coordinate values in each dimension). *SpectrumRegionAxis* is a dense array of these (potentially empty) binary search trees and is used to represent the spectrum of a single peak. The dense array dimension corresponds to ω_5_, i.e., the directly detected dimension

To keep memory consumption within reasonable limits, the 5D version of SSA uses a sparse block structure to represent the five-dimensional spectral region of each peak. Blocks are stored in a self-balancing binary search tree, where the lowest-index point of each block is used as the key. Note that the data structure is sparse only in the indirectly detected dimensions, as shown on Fig. 2. Only the values at spectral points that were marked in step 6 as belonging to the peak are actually stored in the blocks; points that do not belong to the peak are set to zero.

To further reduce the memory requirements, the spectral model of each peak is stored in memory in compressed form, using the *Deflate* algorithm implemented in the *zlib* library, and decompressed only when necessary. This results in over 95% reduction in memory use and output file size. In fact, even when small block sizes are used, a single block in 5D spectra usually contains very few points that actually belong to the peak, and thus consists mostly of zeroes.

**Fig. 3:**
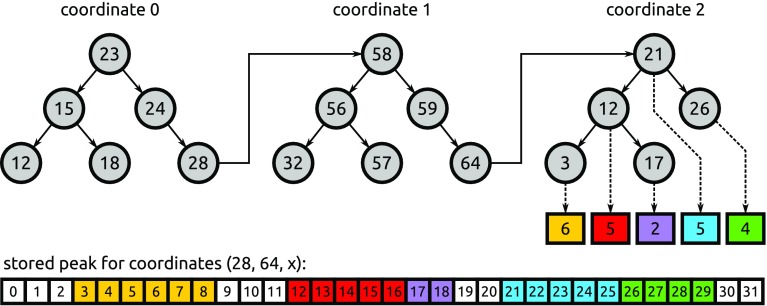
Data structure used for the peak map, shown in the 3D case for simplicity. Each spectral point can belong to at most one peak. The last level of the structure stores lengths of contiguous runs of values belonging to the same peak; different peaks are indicated by *different colours*

A different data structure was used for the five-dimensional peak map, which contains a global information on assignment of spectral points to peaks, without their spectral values. This data is accessed in every step of the signal separation procedure and thus cannot be compressed using a general purpose algorithm. Instead, we used a data structure illustrated in Fig. 3, which stores the lengths of stretches of points belonging to the same peak. Note that it encodes information with the granularity of spectral points instead of blocks. To find a point, each of its coordinates is looked up in a binary search tree that is keyed by the current coordinate and has binary search trees for the next coordinate as its values. At the last level, the tree does not store individual points, but the number of consecutive points belonging to the same peak. As a result, the memory used by the peak map is reduced by approximately 90%, with minimal impact on performance.

In step 7, refining all signals on every iteration may lead to almost no change for large peaks on iteration 3 and later. Therefore, to avoid pointlessly refining signals that no longer change significantly, we stop refining the signal if the last refinement expanded the area of the corresponding peak by less than 1%.

To take advantage of modern multi-core processors, our algorithm uses parallel processing constructs available in OpenMP 3.0. In step 6, blocks are computed asynchronously as the bucket fill algorithm explores the peak, using the task construct. Signal simulation and refinement is also accelerated with OpenMP, exploiting the fact that each (N−1)-dimensional slice of the spectrum across the direct dimension is effectively independent.

Several performance measurements were performed to choose the optimal block size for the sparse spectrum structure and establish the parallel scaling factor of our implementation. The parallel scaling factor is defined as the ratio of single-threaded execution time to multi-threaded execution time multiplied by the number of cores. The optimal scaling factor, achievable with a perfectly parallel algorithm, is by definition 1. We found that a block size of 8 (with each block thus containing 4096 floating point values or 16,384 bytes) is optimal for the 5D case on our hardware; higher block sizes increase memory consumption and processing times. The parallel scaling factor was found to be between 0.68 and 0.72 when using four threads on a 4-core machine (Intel Core i5-2400).

We found that the choice of sampling schedule affects the performance of the algorithm. Poisson disk sampling, which enforces a minimum distance between samples, tends to give low noise in the peak vicinity (Kazimierczuk et al. 2008), but creates ridge-shaped artefacts extending from the peak along the axes of the coordinate system. This requires the use of a higher threshold during the bucket fill step, since the algorithm tends to expand the peak along those ridges into areas with high artefact noise. The peak becomes unbounded and the program eventually exhausts all available memory. By comparison, random sampling with decaying point density gives artefacts distributed more evenly. This allows the bucket fill algorithm to terminate correctly even when a low threshold is set.

## Materials and methods

### NMR spectroscopy

All measurements were performed on a Bruker Avance III HD 800 MHz spectrometer equipped with a cryogenically cooled H-C/N-D TCI probe at 298 K. 3D HNCO (196 min.) was used to prepare input peak list for the Sparse Multidimensional Fourier Transform of 5D data (Kazimierczuk et al. 2009) and 5D SSA. Spectra were processed using SSA implemented in cleaner3d (Stanek and Koźmiński 2010) and cleaner5d programs. Processing software used is available free of charge at http://nmr.cent3.uw.edu.pl/software. All spectra were inspected using the Sparky program (Goddard and Kneller) and manually pick-peaked. Nmrglue Python package was used for visualization purposes (Helmus and Jaroniec 2013).

For the HN(CA)CONH experiment (Kazimierczuk et al. 2010), a pulse sequence with a watergate block (Piotto et al. 1992) was applied. Interscan delay of 1.2 s was set. Only inter-residue correlations were selected by setting the delay for J_NCa_ evolution to 54 ms. In the case of (HACA)CON(CO)CONH experiment (Żerko et al. 2016), the rectangular 180° CO pulses in MOCCA-XY16 were set to 80.26 µs to ensure that Cα nuclei experience an effective 720° rotation during each carbonyl 180° pulse (Felli et al. 2009). 180° pulse repetition period was set to 250 μs and the total mixing time to 400 ms. Interscan delay of 1.3 s was set.

In all experiments four scans were accumulated for each FID, and the acquisition time was set to 85.2 ms (1024 complex points). On-grid sampling schedules randomly drawn from a truncated Gaussian distribution (the standard deviation was set to half of the maximum evolution time) were used in all cases. Other relevant acquisition parameters are summarised in Table 1.

**Table 1:** Experimental parameters of the performed NMR experiments

Experiment	Spectral width [Hz], maximal evolution time [ms], number of TD increments for each indirectly detected dimension			
3D HNCO			^13^C	^15^N
			1200	2700
			106.7	94.8
			256	512
5D HN(CA)CONH	^1^H	^15^N	^13^C	^15^N
	2400	2700	1200	2700
	16.7	26.7	26.7	26.7
	80	144	64	144
5D (HACA)CON(CA)CONH	^13^C	^15^N	^13^C	^15^N
	3000	4000	3000	3000
	32	32	32	32
	192	256	192	192

For processing, a desktop computer with Intel Core i7-3770 CPU and 16 GB RAM was used. For 5D HN(CA)CONH (50 hypercomplex points) and 5D (HACA)CON(CA)CONH (225 hypercomplex points) data sets, processing times of 20′ and of 41 h 23′, respectively, were required.

The peak data extracted from the spectra were stored in 5 MB and 27 MB files, for 5D HN(CA)CONH and 5D (HACA)CON(CA)CONH, respectively. The single cross-section size depending on the desired digital resolution ranges from tens of kB to MBs. The full size 5D spectra (with single zero filling in all dimensions) would require 0.8 TB and 13.5 TB of storage space, respectively.

### Sample preparation

NMR sample of α-synuclein was purchased from Giotto Biotech and contained 1 mM of ^13^C,^15^N-labeled protein in a 20 mM sodium phosphate buffer (pH 6.5), 200 mM NaCl and 10% v/v D_2_O in a standard 5 mm tube.

## Results and discussion

**Fig. 4:**
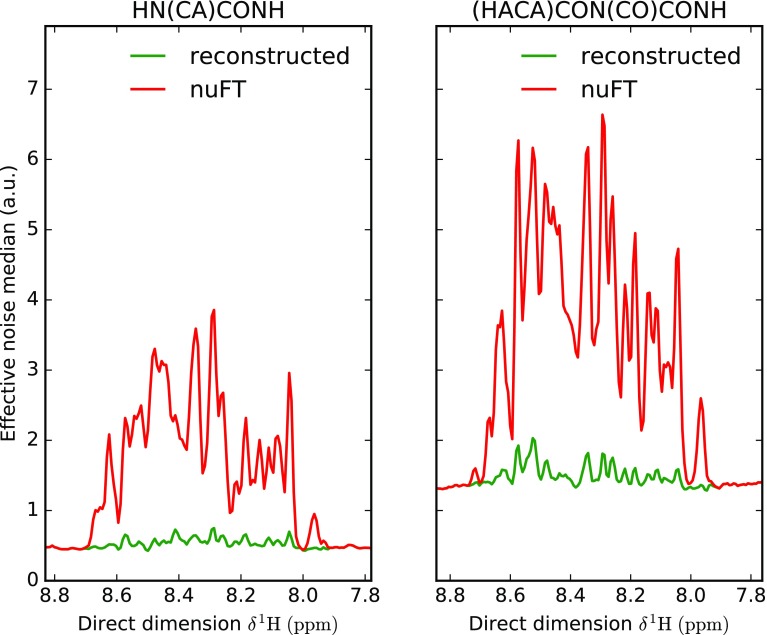
Noise median for 5D HN(CA)CONH (*left*) and 5D (HACA)CON(CO)CONH (*right*) SSA-reconstructed and nuFT spectra with respect to direct dimension δ ^1^H chemical shift

The primary use of 5D experiments is currently the sequential resonance assignment of intrinsically disordered proteins. For this reason, we demonstrate the utility of SSA processing using a sample of human α-synuclein, a paradigmatic small IDP (140 a.a.) involved in neurodegenerative diseases. In this section, we present the reconstruction of two 5D spectra, HN(CA)CONH and (HACA)CON(CO)CONH. The median of noise in reconstructed and nuFT spectra are depicted in Fig. 4. In both cases the effective noise varies significantly from the base line noise in the most populated regions of directly detected dimension (^1^H^N^). The reduction of sampling noise level by SSA is evident, leading to an almost uniform noise level in HN(CA)CONH.

### 5D HN(CA)CONH

The 5D HN(CA)CONH experiment used here was optimized for the detection of sequential correlations only. However, the sensitivity of the experiment is not compromised thanks to the advantageous relaxation properties of IDPs. Obtained spectra contain only one type of correlation peaks, H_i−1_N_i−1_CO_i−1_N_i_H_i_, and are thus straightforward to interpret. Furthermore, this solution naturally reduces the accumulation of sampling artefacts.

**Fig. 5:**
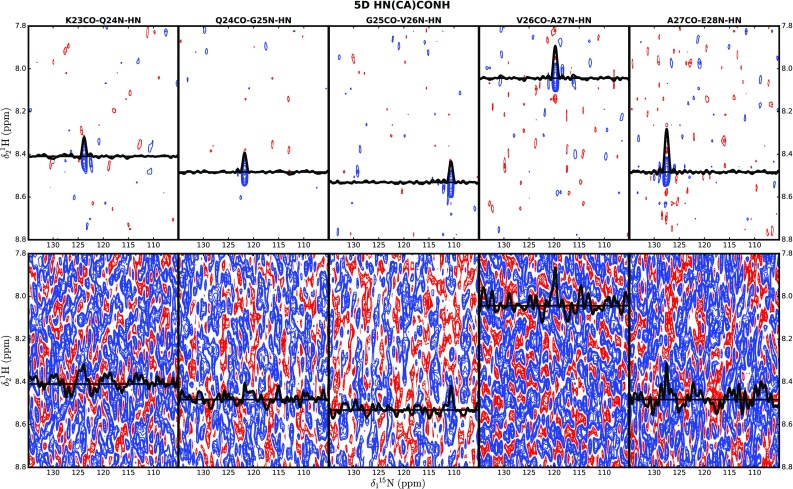
2D cross-sections from 5D HN(CA)CONH for SSA-reconstructed (*top*) and nuFT (*bottom*) spectra. Corresponding pairs of cross-sections were plotted using the same contour levels. Labels of HNCO peaks used for computation of each cross-section are given above the top panel

**Fig. 6:**
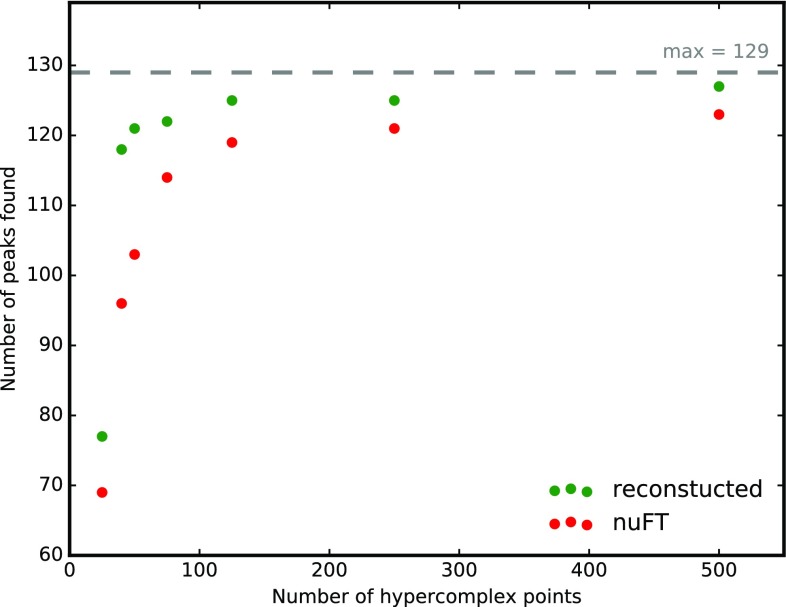
Number of detected peaks in 5D HN(CA)CONH SSA-reconstructed and nuFT spectrum as a function of the number of hypercomplex points used

Partial data sets containing a varying number of hypercomplex points (25, 40, 50, 75, 125, 250 and 500) were prepared and processed using nuFT and SSA. The representative set of five consecutive 2D cross-sections shown in Fig. 5 for a 50-point sampling scheme demonstrates a spectacular improvement in signal-to-noise ratio due to SSA reconstruction. On the contrary, resonances are obscured by artificial noise in nuFT spectra, and their detection requires additional sampling points. All processed cross-sections were inspected for the presence of sequential correlations assuming a minimum S/N of 6; the results are presented in Fig. 6. SSA processing increased spectral quality for all datasets with the most remarkable effect for smaller numbers of sampling points. The 50-point dataset, which corresponds to only a 80 min acquisition, yields the vast majority (94%) of expected peaks, illustrating the potential of SSA to reduce measurement time.

### 5D (HACA)CON(CO)CONH

The 5D (HACA)CON(CO)CONH experiment was designed particularly for sequential resonance assignment and has several salient features. It utilizes the ^3^J(CO–CO) couplings to provide both forward and backward connectivity. The use of TOCSY mixing additionally allows to observe farther correlations, depending on the mixing time. In our dataset, correlations of up to two residues apart were detected. However, peak intensity strongly decreases with the number of relay steps in the TOCSY transfer. Uninformative diagonal peaks are generally the strongest ones. Unless NUS is combined with SSA reconstruction, the presence of artefacts originating from these intense peaks actively diminishes the utility of the experiment.

**Fig. 7:**
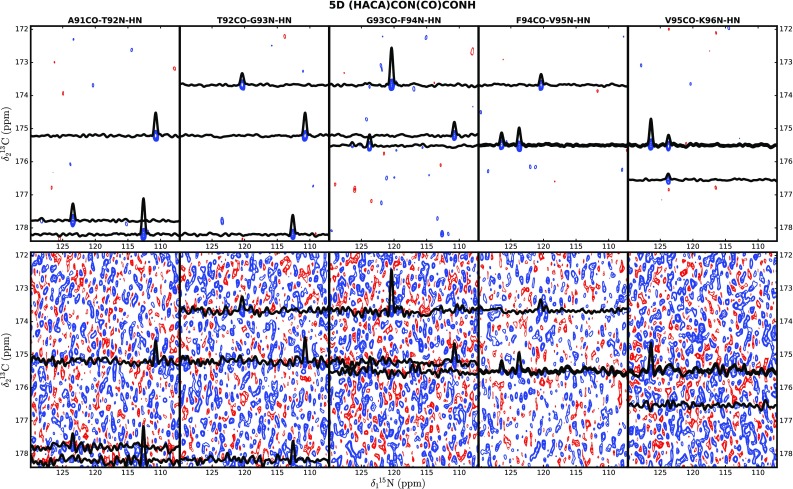
2D cross-sections from 5D (HACA)CON(CO)CONH SSA-reconstructed (*top*) and nuFT (*bottom*) spectra. Corresponding pairs of cross-sections were plotted using the same contour levels. Labels of HNCO peaks used for computation of each cross-section are given above the top panel

**Fig. 8:**
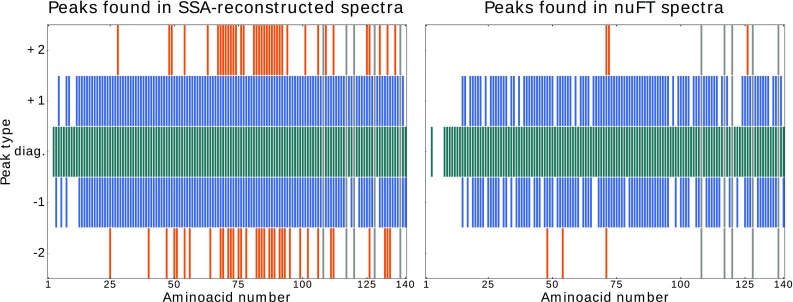
Detected peaks in 5D (HACA)CON(CO)CONH spectra for SSA-reconstructed (*left*) and nuFT (*right*) spectra with respect to amino acid residue number, classified by type (number of relay steps) The colour coding is as follows: blue-green diagonal; *blue* = ±1; *orange* = ±2. Each block represents a single peak. *Grey* blocks represent proline residues with no expected peaks

5D (HACA)CON(CO)CONH was acquired in only 8 h, using 225 hypercomplex sampling points. As before, 3D HNCO peak list was used as input for SSA processing. 2D cross-sections were generated from both SSA–processed and nuFT spectra, and a set of consecutive cross-sections is presented in Fig. 7. All cross-sections were inspected for the presence of diagonal and (+2, +1, −1 and −2) cross-peaks, imposing a minimum S/N of 6. The results are illustrated on Fig. 8 and also summarized in Table 2. SSA-reconstruction substantially increased the number of detectable cross-peaks (+58%), with the most pronounced effect for the weak ±2 correlations, unique to TOCSY experiments.

**Table 2:** Number of detected peaks by type in the 5D (HACA)CON(CO)CONH spectrum

	Diagonal	+1	−1	+2	−2
Reconstructed	133	126	123	37	41
nuFT	129	101	95	6	5

For both of the presented experiments, which demonstrate either low (HN(CA)CONH) or high ((HACA)CON(CO)CONH) dynamic range of peak intensities, SSA efficiently reduces sampling noise. As a result, a spectrum of a given quality can thus be obtained in a shorter time. The computational cost of 5D SSA reconstruction is also reasonable. The computation time on a standard desktop computer ranges from tens of minutes for simple spectra to tens of hours for more complex ones. The primary factors influencing the processing time are the number of acquired data points, digital spectral resolution and number of observed peaks.

We proved that, despite its inherent limitations, SSA yields high-quality five-dimensional spectra. For a majority of high-dimensional spectra NMR of biomolecules, the assumption of well localised spectral features is well fulfilled and allows for local treatment of peak fitting. The computational cost and memory requirements are greatly reduced compared to the methods requiring global optimisations, such as CS and maximum entropy reconstruction. However, for highly resolved 5D spectra even a direct extension of previous SSA would result in unacceptably long computational times. To overcome this problem, we have explicitly imposed the localisation of spectral features in three dimensions to the regions defined by a “root” resonance list. This restricts the search space to an extent that can be processed in a reasonable time. The new reconstruction method outperforms the previously available SMFT, which employs bare nuFT. We would like to note that algorithms such as MDD (Orekhov and Jaravine 2011) or SFFT (Hassanieh et al. 2015) are in principle also suitable for the processing of 5D NUS data, however, no implementation is so far available and thus one cannot compare the quality of reconstructions in terms of sensitivity, convergence and sampling thresholds. While α-synuclein used in this study is a relatively small IDP and thus spectral overlap is quite limited and possible to overcome using 3D and 4D spectroscopy, larger IDPs obviously exhibit more severe spectral crowding, which necessitates the acquisition of 5D experiments. At the same time, peak congestion leads to elevated sampling noise, therefore increased gains from spectral reconstruction for larger IDPs are to be expected.

## Conclusions

We presented for the first time an efficient implementation and application of a reconstruction algorithm of five-dimensional NMR spectra from sparsely sampled data with an active suppression of NUS artefacts. The new algorithm enables detection of less intense peaks in the presence of strong signals, which are observed for an important class of experiments based on TOCSY transfer. Alternatively, it can be employed to reduce experimental time required for the acquisition of certain 5D spectra that show a lower number of peaks and/or feature limited range of peak intensities. The method yields high-quality, well resolved spectra in the challenging case of intrinsically disordered proteins.

## References

[CR1] Baias M, Smith PES, Shen K, Joachimiak LA, Żerko S, Koźmiński W, Frydman J, Frydman L (2017). Structure and dynamics of the huntingtin exon-1N-terminus: a solution NMR perspective. J Am Chem Soc.

[CR2] Coggins BE, Zhou P (2008). High resolution 4-D spectroscopy with sparse concentric shell sampling and FFT-CLEAN. J Biomol NMR.

[CR3] Coggins BE, Venters RA, Zhou P (2010). Radial sampling for fast NMR: concepts and practices over three decades. Prog Nucl Magn Reson Spectrosc.

[CR4] Coggins BE, Werner-Allen JW, Yan A, Zhou P (2012). Rapid protein global fold determination using ultrasparse sampling, high-dynamic range artifact suppression, and time-shared NOESY. J Am Chem Soc.

[CR5] Felli IC, Pierattelli R, Glaser SJ, Luy B (2009). Relaxation-optimised Hartmann–Hahn transfer using a specifically Tailored MOCCA-XY16 mixing sequence for carbonyl–carbonyl correlation spectroscopy in 13 C direct detection NMR experiments. J Biomol NMR.

[CR6] Freeman R, Kupče E (2012). Concepts in projection-reconstruction. Top Curr Chem.

[CR7] Goddard TD, Kneller DG (2000). SPARKY 3.

[CR8] Haba NY, Gross R, Nováček J, Shaked H, Židek L, Barda-Saad M, Chill JH (2013). NMR determines transient structure and dynamics in the disordered C-terminal domain of WASp interacting protein. Biophys J.

[CR9] Hassanieh H, Mayzel M, Shi L, Katabi D, Orekhov VY (2015). Fast multi-dimensional NMR acquisition and processing using the sparse FFT. J Biomol NMR.

[CR10] Helmus JJ, Jaroniec CP (2013). Nmrglue: an open source Python package for the analysis of multidimensional NMR data. J Biomol NMR.

[CR11] Hiller S, Wider G (2012). Automated projection spectroscopy and its applications. Top Curr Chem.

[CR33] Hiller S, Wasmer C, Wider G, Wüthrich K (2007). Sequence-specific resonance assignment of soluble nonglobular proteins by 7D APSY-NMR spectroscopy. J Am Chem Soc.

[CR12] Högbom J (1974). Aperture synthesis with a non-regular distribution of interferometer baselines. Astron Astrophys Suppl Ser.

[CR13] Holland DJ, Bostock MJ, Gladden LF, Nietlispach D (2011). Fast multidimensional NMR spectroscopy using compressed sensing. Angew Chem Int Ed Engl.

[CR14] Kazimierczuk K, Orekhov VY (2011). Accelerated NMR spectroscopy by using compressed sensing. Angew Chem Int Ed Engl.

[CR15] Kazimierczuk K, Zawadzka A, Koźmiński W (2008). Optimization of random time domain sampling in multidimensional NMR. J Magn Reson.

[CR16] Kazimierczuk K, Zawadzka A, Koźmiński W (2009). Narrow peaks and high dimensionalities: exploiting the advantages of random sampling. J Magn Reson.

[CR17] Kazimierczuk K, Zawadzka-Kazimierczuk A, Koźmiński W (2010). Non-uniform frequency domain for optimal exploitation of non-uniform sampling. J Magn Reson.

[CR18] Kazimierczuk K, Misiak M, Stanek J, Zawadzka-Kazimierczuk A, Koźmiński W (2012). Generalized Fourier transform for non-uniform sampled data. Top Curr Chem.

[CR19] Mobli M, Hoch JC (2008). Maximum entropy spectral reconstruction of non-uniformly sampled data. Concepts Magn Reson A.

[CR20] Nagayama K, Bachmann P, Wüthrich K, Ernst RR (1978). The use of cross-sections and of projections in two-dimensional NMR spectroscopy. J Magn Reson (1969).

[CR21] Narayanan RL, Duerr HN, Bilbow S, Biernat J, Mendelkow E, Zweckstetter M (2010). Automatic assignment of the intrinsically disordered protein Tau with 441-residues. J Am Chem Soc.

[CR22] Nowakowski M, Saxena S, Stanek J, Żerko S, Koźmiński W (2015). Applications of high dimensionality experiments to biomolecular NMR. Prog Nucl Magn Res Spectrosc.

[CR23] Orekhov VY, Jaravine VA (2011). Analysis of non-uniformly sampled spectra with multi-dimensional decomposition. Prog Nucl Magn Res Spectrosc.

[CR24] Piai A, Calçada EO, Tarenzi T, Grande AD, Varadi M, Tompa P, Felli IC, Pierattelli R (2016). Just a flexible linker? The structural and dynamic properties of CBP-ID4 revealed by NMR spectroscopy. Biophys J.

[CR25] Piotto M, Saudek V, Sklenář V (1992). Gradient-tailored excitation for single-quantum NMR spectroscopy of aqueous solutions. J Biomol NMR.

[CR34] Robin M, Delsuc MA, Guittet E, Lallemand JY (1991). Optimized acquisition and processing schemes in three-dimensional NMR spectroscopy. J Magn Reson (1969).

[CR26] Stanek J, Koźmiński W (2010). Iterative algorithm of discrete Fourier transform for processing randomly sampled NMR data sets. J Biomol NMR.

[CR27] Stanek J, Augustyniak R, Koźminski W (2012). Suppression of sampling artefacts in high-resolution four-dimensional NMR spectra using signal separation algorithm. J Magn Reson.

[CR28] Szyperski T, Wider G, Bushweller JH, Wüthrich K (1993). Reduced dimensionality in triple-resonance NMR experiments. J Am Chem Soc.

[CR29] Tang J, Norris JR (1988). LP-ZOOM, a linear prediction method for local spectral analysis of NMR signals. J Magn Reson.

[CR30] Ying J, Delaglio F, Torchia DA, Bax A (2016). Sparse multidimensional iterative lineshape-enhanced (SMILE) reconstruction of both non-uniformly sampled and conventional NMR data. J Biomol NMR.

[CR31] Żerko S, Koźmiński W (2015). Six- and seven-dimensional experiments by combination of sparse random sampling and projection spectroscopy dedicated for backbone resonance assignment of intrinsically disordered proteins. J Biomol NMR.

[CR32] Żerko S, Byrski P, Włodarczyk-Pruszyński P, Górka M, Ledolter K, Masliah E, Konrat R, Koźmiński W (2016). Five and four dimensional experiments for robust backbone resonance assignment of large intrinsically disordered proteins: application to Tau3x protein. J Biomol NMR.

